# Heads in the sand may leave old age psychiatry looking foolish and vulnerable: Commentary on . . . A memory clinic *v.* traditional community mental health team service

**DOI:** 10.1192/pb.bp.114.048215

**Published:** 2015-02

**Authors:** David Jolley

**Affiliations:** 1 PSSRU, The University of Manchester

## Abstract

Dementia has been recognised as a major challenge to health, social care and economies. Research by Rubinsztein and colleagues, in this issue, has compared the services provided by memory clinics with those of traditional community mental health team services. They conclude that memory clinics offer a more comprehensive and multidisciplinary service at no extra cost. Here I will question some of their findings and highlight the importance of better continuity of care between primary and secondary services.

These are heady times for the dementia lobby. London’s G8 Conference on dementia was the latest in a series of national and international ‘think-ins’ that has seen the condition emerge from the shadows of denial and neglect to be recognised as the single most significant challenge to health and social care, economies and personal philosophy now and for the predictable future.^1^

*The Guardian*’s letters page and a thoughtful article from Richard Ashcroft laid bare the realities of our situation.^2,3^ Awareness of dementia has been improved, but are services being made available to help people affected by it and are the research initiatives producing better lives and reducing stress associated with the condition? Richard Ashcroft’s mother-in-law received a diagnosis and was discharged after two contacts by ‘old age psychiatry’, leaving her, her family and general practitioner to feel left adrift in a sea made no-less frightening by having acquired a label.

## Rubinsztein *et al*’s research

Judy Rubinsztein and her colleagues^4^ provide an interesting and important description and analysis of what happens when someone with a memory problem is referred by primary care for specialist assessment and advice. They compare the experiences and costs associated with a memory clinic, the model that has become the Holy Grail of assessment through the National Dementia Strategy,^5^ and assessment with less formality by a community mental health team (CMHT). Referral rates were similar: 5 per 1000 of the over 64-year-old population per annum. The CMHT patients were older, they were less impaired and were seen more quickly after referral. The diagnostic spectra were similar. Neither service was bedevilled by a long waiting list such as is described by many floundering services.^6^ Stakeholders were happy with both systems but measures that equate quality with assessment by more than one profession, and make use of formalised paper protocols and checklists, prefer the memory clinic model: memory clinic patients were twice as likely to receive a copy of the letter summarising the findings and plans for their future care.

The increased ‘quality’ attributed to the memory clinic is said to be achieved at no greater, actually lesser, cost. Yet, we might wonder how useful all those paper scaled measures are and many will question the costings: digging deeper we find that half of patients referred to the CMHT service were seen only once, and by a consultant in their own homes. The paper judges this to be poor practice, yet this is not reflected in stakeholder views. It might alternatively be viewed as an elegant and efficient approach that reserves multidisciplinary assessments at the clinic, which are time-consuming, less convenient and more costly for patients and carers, for people with more complex presentations. The memory clinic model might be construed as: ‘one-size-fits-all’ with everyone attending the clinic, 22/33 having two to six contacts. This is the sort of consideration that makes people question the advantages of clinic-based services. The differential that deems the CMHT model more expensive relates exclusively to travel costs, where the high salary of consultants who are travelling and one outlier who was visited eleven times, load the CMHT pricing. This is a brave and important attempt to capture costs and relate these to activities and effectiveness. It leaves us to reflect how difficult a task this is.

The paper opens a fascinating window on what actually happens in this world of dementia care. A total of 35% per cent of people ‘eligible’ for cholinesterase inhibitors did not receive them. This is the reality and gives a degree of balance to criticism of the UK for its relatively low rate of prescribing these substances:^7^ even when assessed, not every patient will accept such treatment, others will encounter side-effects or become disabused. One wonders what is happening in those countries that report prescriptions to near 100% of the predicted prevalence of Alzheimer’s disease.

Three CMHT patients (10%) were retained for further care, but only 1 of 33 in the memory clinic was directed to their CMHT. One patient (out of 66) received cognitive stimulation therapy and three saw a neuropsychologist. The memory clinic is applauded for ‘signposting’ more patients to other services: third sector, social services or benefits. Overall, Richard Ashcroft might be forgiven for feeling that not much of substance is evident after the initial flurry.

## Identifying weakness in existing services

The arguments in favour of including specialist memory services within the spectrum offered by old age psychiatry and other disciplines are strong, cogent and widely accepted.^8^ With great respect, however, both models described here are failing. Their referral rates are such that they cannot close the gap that still exists between predicted prevalence and diagnosed prevalence.^9^ There is no increase in referrals associated with the memory clinic arrangement, nor are the patients seen by that clinic earlier in the course of their dementia (as measured using the Mini-Mental State Examination). Tellingly, neither makes a tangible contribution to the continuity of care that patients, families and colleagues in other agencies respect and expect.

Variations that simply replace doctors with cheaper nurses, rate per hour^10^ may not be more cost-effective. They are more likely to rely on standardised protocols with inclusion/exclusion criteria designed to be risk-avoidant and limit workload rather than respond to patient need.^11^ Lessons from the 10/66 studies and initiatives encourage the use of low-tech, clinically competent approaches with training and support to local healthcare agents.^12,13^

In countries with established large populations of older people, including the UK, the realisation is that we must bring specialist skills into primary care so that people with dementia can be assessed, treated and supported by a competent local team that knows them as whole people with multiple strengths and multiple weaknesses associated with a range of pathologies.^14–16^ Models that do this achieve referral rates more than twice those reported in East Anglia, sustain patients, carers and primary care colleagues throughout the journey of dementia and other frailties before death, and reduce expenditure on secondary health care.^17^

Despite the rhetoric of ‘war on dementia’, and exposure of the myth of the dependency ratio,^18^ actual service support for older people, including those with dementia, has been reduced by 30% in this country.^19^ Populist politicians are given column space to stir up unjustifiable resentment against old people.^20^

Psychiatrists and their colleagues need to remain clear-headed, open and honest as advocates and providers for people with dementia and their families. We are learning what works and is affordable and this is what matters.

## Conclusions

Rubinsztein and her colleagues have done us good service in dissecting and comparing the innards of clinic-based and community-based memory services. They have answered some questions and opened others to be pursued, which is all to the good; but we must lift our heads from the sand of what we have been doing within the comfort and discomfort of secondary care to work across the false border that is assumed between primary care and secondary care. There is little to be gained from a well-made diagnosis unless it is part of a meaningful, continuing process of care for the patient and their caring family.
